# Using Evidence-based Community and Behavioral Interventions to Prevent Skin Cancer: Opportunities and Challenges for Public Health Practice

**Published:** 2005-03-15

**Authors:** Karen Glanz, Mona Saraiya

**Affiliations:** Department of Behavioral Sciences and Health Education, Rollins School of Public Health, Emory University; Division of Cancer Prevention and Control, Centers for Disease Control and Prevention, Atlanta, Ga

## Introduction

Skin cancer is the most common cancer in the United States and is increasing in incidence (1). In 2004, more than 1 million people were expected to be diagnosed with squamous cell or basal cell carcinoma, and more than 2200 deaths were expected (2). Another 54,200 people were estimated to be diagnosed with melanoma, the most lethal of all skin cancers, and 7600 persons were expected to die from that disease during 2004. High levels of exposure to ultraviolet radiation (UVR) increase the risk of all three major forms of skin cancer, and approximately 65% to 90% of melanomas are caused by UVR exposure. Other risk factors for skin cancer include having fair skin, hair, and eyes; growing up closer to the equator; and having a large number of moles or nevi (3).

Fortunately, skin cancer is one of the most preventable cancers. State and local health departments can play an important role in preventing skin cancer by developing population-based programs to prevent the disease; assuring sun-safe environments and policies; and regulating exposure where appropriate. Behaviors that reduce risk include limiting or minimizing exposure to the sun during midday hours; wearing protective clothing; and using a broad-spectrum sunscreen when outside (3).

The Task Force on Community Preventive Services conducted an evidence-based review of the efficacy of interventions for sun protection in varied segments of the population across various settings (4,5). Reviewers examined the methodology of identified studies to see whether their design was suitable and their execution good enough to be included in the Task Force's review and also to inform the later determination of whether the evidence was sufficient to recommend a particular intervention (6,7). Given the increasing emphasis on basing policy and practice on evidence, public health leaders and practitioners should be familiar with this evidence review, its findings, and its implications for policy and practice.

This paper summarizes the state of knowledge about the effectiveness of interventions to reduce UVR exposure among various groups to prevent skin cancer and suggests strategies and resources for translating the evidence into action to improve population health.

## State of the Evidence in Settings Most Influenced by Public Health Agencies

### Methods

The Task Force on Community Preventive Services conducted systematic evidence reviews of the effectiveness of interventions for reducing UVR exposure to prevent skin cancer, using rigorous but standard methodology developed for the *Guide to Community Preventive* *Services* (*Community Guide*) (6) and methodology specific to this review (5). These reviews examined behavioral, educational, policy, and environmental strategies for changing behaviors to reduce skin cancer risk (5). In establishing the criteria for the evidence review, the task force accepted several premises: 1) exposure to sun helps cause skin cancer; 2) covering up and avoiding exposure to UVR plays a protective role; and 3) an outcome of using sunscreen by itself is not an indicator of intervention effectiveness (4).

A conceptual model, or analytic framework, was developed to show the relationship of the interventions to relevant intermediate outcomes (e.g., knowledge, attitudes, intentions regarding sun-protective behaviors) to actual behaviors and the prevention of skin cancer. Outcome data extracted from the studies were aligned with the analytic framework to answer research questions.

Key outcome targets identified in the analytic framework were improvements in knowledge, attitudes, and intentions relative to reducing UVR exposure or increasing protection from the sun; changes in exposure and protection; reduction of sunburn; and changes in policies and environments aimed at reducing exposure (e.g., limiting exposure during peak sun hours, increasing shade, providing sunscreen). The review team considered sunscreen use to be a secondary outcome because, although sunscreens prevent sunburn, their role in preventing melanoma has not been unequivocally shown (8,9). Also, although none of the studies identified measured incidence of precancer, nevi, photodamage, or skin cancer, the review team assumed that behavioral changes and reduction of sunburn, if achieved, would lead to lower rates of cancer (5).

To give a positive recommendation, the task force requires at least two high-quality studies showing positive effects. The evidence reviews covered nine categories of interventions. Six focused on distinct settings: health care and health care providers, the workplace, recreation/tourism, secondary schools and colleges, primary schools, and child care centers. The other three categories focused on a target population (e.g., children's parents and caregivers) or broad interventions (e.g., media campaigns, community-wide multicomponent interventions). The focus was strictly on prevention, not early detection.

### Main findings

Of particular interest to health departments are the findings for settings in which health departments have advisory, collaborative, or regulatory roles: day care, recreation centers, primary schools, work sites, community-wide programs, and media campaigns. These findings are summarized here.

In two settings, evidence was sufficient to recommend interventions: primary schools and recreation/tourism. Educational and policy interventions in primary schools had sufficient evidence of increasing children's covering-up behavior — specifically, wearing protective clothing and hats. Approaches included interactive classroom and take-home activities about sun protection, brochures for parents, and a working session to develop plans and policies for sun protection. These approaches provided sufficient evidence of improvement in covering-up behavior, with a median relative increase of 25% across six studies of good quality (the Appendix provides definition of relative increase). Evidence was insufficient to determine the effectiveness of interventions in improving other behaviors, such as avoiding the sun, because of inconsistent results; evidence was also not sufficient to determine effectiveness in decreasing sunburns because there was only one study, which was limited in design and execution.

Evidence was also sufficient for the effectiveness of interventions in recreation/tourism settings, specifically for increasing adult covering-up behavior, with a median net increase of 11.2% across five studies. These interventions included one or more of these strategies: training in sun safety and role modeling by outdoor recreation staff and lifeguards; providing lessons in sun safety, interactive activities, and programs for parents; increasing available shaded areas; providing sunscreen and educational brochures; and offering point-of-purchase prompts. In contrast, intervention studies yielded insufficient evidence to determine effectiveness in affecting children's sun-protective behavior; results were inconsistent.

The Task Force on Community Preventive Services found insufficient evidence on which to make recommendations for or against interventions to reduce exposure to UVR in the following settings and populations: child care centers, secondary schools and colleges, recreation/tourism settings for children, occupational settings, media campaigns alone, and community-wide multicomponent interventions (4). A finding of insufficient evidence, however, does not suggest that an intervention does not or cannot work; rather, it indicates that the available evidence base was insufficient in quality or quantity to make a determination (10). Furthermore, many of the studies had multiple components that could not be evaluated separately (4); some strategies for which effectiveness was not evaluated independently might be part of an effective community program.

## Translating Evidence Into Action

The findings of the evidence review for the *Community Guide *on interventions to reduce UVR exposure have an important place in evidence-based decision making among public health officials. They should be considered when identifying legislative and policy approaches that support prevention and in developing research agendas (10,11). While evidence-based policy and practice is an increasing priority, it is equally necessary to mobilize community partnerships to identify and address health problems (12).

One evaluation of the process of disseminating earlier *Community Guide* findings found that city and county health department program directors believed that rigorous information about the effectiveness of interventions was important, but the directors noted that evidence-based recommendations alone do not assure the implementation of effective interventions (13). These evidence reviews clearly fill a gap, however: an analysis of the data-based planning activities of state health agencies in the mid-1990s found that there were few useful sources of data on proven preventive interventions and how to implement them (14).

Efforts to translate *Community Guide* evidence review into action should use local data, the recommendations, and resources available from federal agencies, voluntary health organizations, and academic sources. In particular, public health planners and program directors can benefit from several program models and ready-made tools for program planning, implementation, and evaluation in the prevention of skin cancer. The "Guidelines for School Programs to Prevent Skin Cancer" (15) can be used to help shape policy and curricular interventions. The Centers for Disease Control and Prevention offers free online resources for skin cancer prevention and education (16), and the Cancer Control PLANET Web site includes a step-by-step model for effective planning of skin cancer control (17).

The National Comprehensive Cancer Control Program provides a model, a framework, and funding to develop state cancer prevention plans. The planning process involves leadership from state health departments using data-driven priorities and multisectoral cooperation (18,19). A review of available state cancer plans shows a range of objectives and actions, including 1) plans to determine the prevalence of sunburn using data from national surveys such as the National Health Interview Survey or state-based data from the Behavior Risk Factor Surveillance System (20); 2) the establishment of objectives related to awareness, policy change, and reduction of sunburns (21) and 3) detailed analyses of incidence and trends for melanoma in population subgroups, analysis of barriers, and clear goals and action plans (22).

Research and evaluation in states and local communities are important to the continuing growth of the evidence base in preventing skin cancer and can be accomplished by health department personnel with academic and other public health system partners (12). In Hawaii, a survey of elementary school principals showed that most were aware of the risks of excess UVR exposure, but few policies were in place; still, these principals were receptive to statewide leadership for prevention (23). In Georgia, a statewide cancer control program focused initially on breast and cervical cancer, but it planned to expand into preventing skin cancer (24). In addition, a Maine project to prevent skin cancer using components from various well-researched strategies (25) could provide useful information to other states by adding a structured program evaluation.

## Conclusion

Both opportunities and challenges emerge from the evidence review on interventions to prevent skin cancer conducted for the *Community Guide.* First, readers should note that the absence of sufficient data to prove the efficacy of primary prevention efforts in specific settings or subpopulations is not proof of inefficacy. Rather, the findings reveal the need for additional evaluation of efforts to achieve primary prevention. Public health agencies have room for improvement and involvement. Opportunities for involvement include taking a leadership role in developing policies and regulations to reduce UVR exposure, especially among children; working with the media to communicate consistent and effective messages about sun protection; and engaging with the private sector to encourage adoption of protections and policies for outdoor workers.  

Public health departments also have opportunities to contribute to areas in which there is sufficient evidence that strategies to prevent skin cancer have been effective. Divisions charged with preventing chronic diseases can work with schools and recreational settings by helping them to set policies and adopt prevention curricula. The credibility of school and recreation administrators as community leaders can enable them to be powerful communicators about how skin cancer may affect their populations.

Although the *Community Guide *does not show that interventions to prevent skin cancer are useful in many settings, it does support an effect in primary schools and outdoor recreation. These findings suggest that public health agencies should allocate resources to primary schools and outdoor recreation while refining and confirming the efficacy of interventions in other settings. Ultimately, the importance of the *Community Guide* evidence review "will be determined by its impact on enhancing health and quality of life in communities" (26).

## Appendix

**Table:** Summary Effect Measures^a^

	**Before-and-after-only design**	**Study with comparison group** **(RCT, cohort design, nonrandomized trial)**
Absolute effect measure:	post − pre	ΔI − ΔC
Relative effect measure:	(post − pre)/pre × 100	(ΔI/Ipre − ΔC/Cpre) × 100

a RCT indicates randomized controlled trial; I indicates intervention; C indicates control; Δ indicates change.
